# Expressed Sequence Tags from the oomycete *Plasmopara halstedii*, an obligate parasite of the sunflower

**DOI:** 10.1186/1471-2180-7-110

**Published:** 2007-12-06

**Authors:** Mohamed Fouad Bouzidi, Francis Parlange, Paul Nicolas, Said Mouzeyar

**Affiliations:** 1 UMR 1095 INRA-UBP "Amélioration et Santé des Plantes", Université Blaise Pascal, 24, Avenue des Landais 63177 Aubière Cedex, France

## Abstract

**Background:**

Sunflower downy mildew is a major disease caused by the obligatory biotrophic oomycete *Plasmopara halstedii*. Little is known about the molecular mechanisms underlying its pathogenicity. In this study we used a genomics approach to gain a first insight into the transcriptome of *P. halstedii*.

**Results:**

To identify genes from the obligatory biotrophic oomycete *Plasmopara halstedii *that are expressed during infection in sunflower (*Helianthus annuus *L.) we employed the suppression subtraction hybridization (SSH) method from sunflower seedlings infected by *P. halstedii*. Using this method and random sequencing of clones, a total of 602 expressed sequence tags (ESTs) corresponding to 230 unique sequence sets were identified. To determine the origin of the unisequences, PCR primers were designed to amplify these gene fragments from genomic DNA isolated either from *P. halstedii *sporangia or from *Helianthus annuus*. Only 145 nonredundant ESTs which correspond to a total of 373 ESTs (67.7%) proved to be derived from *P. halstedii *genes and that are expressed during infection in sunflower. A set of 87 nonredundant sequences were identified as showing matches to sequences deposited in public databases. Nevertheless, about 7% of the ESTs seem to be unique to *P. halstedii *without any homolog in any public database.

**Conclusion:**

A summary of the assignment of nonredundant ESTs to functional categories as well as their relative abundance is listed and discussed. Annotation of the ESTs revealed a number of genes that could function in virulence. We provide a first glimpse into the gene content of *P. halstedii*. These resources should accelerate research on this important pathogen.

## Background

Sunflower downy mildew is a major disease caused by the Oomycete *Plasmopara halstedii *(Farl.) Berl et de Toni. The first physiological race of this obligate parasitic oomycete has been identified by Zimmer in North America and Europe [1]. Both in compatible and incompatible interactions, host penetration occurs at the lower part of the hypocotyl [2]. Usually, about thirteen days after artificial infection of susceptible lines, the parasite invades almost all the plant tissues and is present in the cotyledons, epicotyls and leaves. In contrast, from the fifth days and onwards, a hypersensitive-like reaction develops within the hypocotyl of resistant lines and in many cases, the parasite's growth is arrested before it reaches the cotyledons [2,3]. Molecular analysis showed that the resistance could be associated with an unusual delayed hypersensitive reaction and a systemic acquired response that take place inside the hypocotyls with the seedlings showing no apparent symptoms [3].

The establishment of the disease or the resistance is the result of the expression of defence genes in the host and virulence or pathogenicity genes in the parasite. In the sunflower, some defence-related genes whose expression varied in compatible and incompatible interactions have been characterized [3,4]. In contrast, genes from *P. halstedii *potentially involved in the infectious process have not been reported yet. This may be explained by the obligate nature of the development of *P. halstedii *on its host.

Since completion of the *Saccharomyces cerevisiae *genome [5], progress on the Genome sequence information and expressed sequence tag (EST) collections from several other parasitic and symbiotic fungi that infect humans, other animals and plants are also becoming more widespread [6,7]. More recently, the whole genome sequences of *Phytophthora ramorum *and *Phytophthora sojae*, two major oomycetes pathogens have been reported [8], providing the framework for comparative genomics studies [9] or the identification of specific gene families potentially implicated in the infectious process [10]. Similarly, the availability of the whole genome sequence of *Hyaloperonospora parasitica *genome should help in the discovery of similar genes in the other oomycetes [11]. The EST (Expressed Sequence Tags) approach represents a relatively simple procedure for finding genes and generating information about their expression in organisms with no genetic research history [12]. For example, in *Blumeria graminis*, 4908 ESTs representing 1669 individual genes have been obtained by sequencing clones from two cDNA libraries from germinating and ungerminated conidia [13]. In a different work, van der Biezen *et al.*[14] used the cDNA-AFLP strategy to clone 10 cDNA fragments from the obligatory biotrophic oomycete *Hyaloperonospora parasitica *(formerly *Peronospora parasitica *(Fr.)) during infection in *Arabidopsis thaliana*. Similarly, Casimiro *et al .*[15] used DD-PCR to identify 21 ESTs from *H. parasitica *infecting *Brassica oleracea*.

Here we report the cloning and analysis of 602 EST obtained by Subtractive Suppression Hybridization PCR [16] from sunflower seedlings infected by *P. halstedii*. In addition, the origin of these ESTs was checked by PCR using specific primers and genomic DNA isolated from *P. halstedii *or from sunflower.

## Results

### Expressed sequence tags analysis

After two rounds of subtraction hybridization, cDNAs were cloned into pGemT-easy vector and the bacteria arrayed in 96 well plates. To estimate the average size of the obtained clones, 40 clones were randomly chosen and their inserts were amplified using the SP6 and T7 universal primers present on the vector. The amplification products were then separated by agarose gel electrophoresis. The estimated sizes were between 400 and 800 bp with an average of about 500 bp. Subsequently, 602 clones were randomly chosen and single pass sequenced using the T7 universal primer. The length of good quality sequences was on average between 400 and 500. 51 sequences were of a poor quality or too short (<100 bases) thus were excluded from further analysis.

The remaining 551 sequences were compared to each other using the BlastN program [17] to identify overlapping sequences and assembled into contigs using the CAP3 program [18]. One hundred fifty three clones out of 551 were found as singletons and the remaining 398 clones formed 77 contigs containing at least 2 clones. Thus 230 unisequences were present in 551 cDNA clones analysed. The relative abundance of identical clones within the collection is shown in Table 1. The number of clones per contig ranged from 2 to 78. Half of the sequences were either unique or formed contigs containing 2 or 3 clones. Two contigs only contained respectively 52 and 78 clones, which represent approximately 24% of the total (cf. Table 1).

**Table 1:** Redundancy of EST clones and number of duplicates

**Redundancy of EST clones**	1	2	3	4	5	6	7	8	9	10	11	12	25	55	78
**Number of clones**	153	35	19	8	4	3	3	0	0	1	0	1	1	1	1

### PCR amplification and origin of ESTs

**Figure 1:**
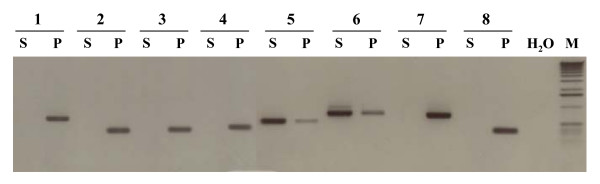
**Examples of PCR amplification of ESTs originatingeither from sunflower (S) or from *Plasmopara halstedii *(P)**. Each primer pair was tested using DNA from either sunflower (**S**), or *Plasmopara halstedii *sporangia (**P**). The ESTs tested here are: **1**, HSP 70 [GenBank:CB174638]; **2**, Pyruvate kinase [GenBank:CB174571]; **3**, Superoxyde dismutase [GenBank:CB174636]; **4**, Proteasome 26 S beta subunit [GenBank:CB174617]; protein; **6**, Asparagine synthase; **7**, Elicitor [GenBank:CB174646]; **8**, Cyclophilin B [GenBank:CB174647]. **M**: molecular marker. **H_2_0**: negative control. The PCR products were separated on 1.5% agarose gel.

Because the 230 unisequences could originate either from *P. halstedii *or represent induced genes in sunflower, PCR primers were designed to amplify these gene fragments from DNA isolated either from *P. halstedii *sporangia or from *Helianthus annuus*. All the 230 primer pairs tested amplified fragments either from *P. halstedii *DNA or *from Helianthus annuus *DNA; 145 primer pairs amplified fragments only from *P. halstedii *DNA, indicating that these ESTs were derived from *P. halstedii *genes that are expressed during infection in sunflower. Figure 1 shows an example of PCR amplifications from DNA isolated from *P. halstedii *sporangia or from *Helianthus annuus*.

### Homology search

To identify homologs of the 145 ESTs derived from *P. halstedii*, each EST sequence was queried against the NCBI non-redundant protein database using the BLASTX algorithm [17]. Table 2 shows the proportion of EST sequences with no significant similarity to known protein sequences (> E^-05^), significant similarity (E^-05 ^to E^-20^), or highly significant (< E^-20^). A total of 89 non-redundant sequences, which correspond to 60% of the 145 ESTs obtained, showed significant (< E^-05^) homology to sequences in the NCBI database and thus were retained for functional classification (Table 3).

**Table 2:** Frequency of the resulting BlastX *P*-values of *Plasmopara halstedii *ESTs

***P*-Value**	**Number of nonredundant EST**	**Number of total EST**
> E^-05^	56	95
E^-05 ^to E^-20^	43	56
< E^-20^	46	222
Total	145	373

**Table 3:** Putative identification of *Plasmopara halstedii *cDNA confirmed by PCR

**GenBank: Accession Number (a)**	**2. Putative identification**	**Number**	***P-*Value**	**Species (b)**
**Cell metabolism**				
CB174570	Isocitrate dehydrogenase	1	1e-55	*Nicotiana tabacum*
CB174571	Pyruvate kinase	1	5e-035	*Achlya bisexualis*
CB174572	Fructokinase	3	9e-10	*Pediococcus pentosaceus*
CB174573	Methylmalonate-semialdehyde dehydrogenase	1	8e-019	*Stigmatella aurantiaca*
CB174574	Glucose transporter	1	5e-05	*Gallus gallus*
CB174575	Glucose-6-phosphate isomerase	1	3e-073	*Phytophthora infestans*
CB174576	F1-ATP synthase subunit B	1	8e-16	*Psychrobacter arcticus*
CB174577	ATP synthase subunit C	1	4e-19	*Candidatus Pelagibacter*
CB174578	F1-ATP synthase beta subunit	1	4e-97	*Arabidopsis thaliana*
CB174579	NADH dehydrogenase 24 kDa subunit	1	3e-59	*Rattus norvegicus*
CB174580	Quinone-oxidoreductase	1	8e-21	*Oryza sativa*
CB174581	NDP Kinase	6	5e-50	*Homo sapiens*
**Cell structure**				
CB174582	Actin	7	2e-31	*Pythium irregulare*
CB174583	Potassium channel beta subunit	1	1e-46	*Phytophthora infestans*
CB174584	Transport protein SEC61 gamma subunit	3	2e-19	*Mus musculus*
CB174585	Transportin	1	8e-08	*Drosophila melanogaster*
CB174586	Aquaporin	1	1e-024	*Sparus aurata*
CB174587	V-type ATPase	1	7e-08	*Pleurochrysis carterae*
**Protein metabolism**				
CB174714	40S ribosomal protein S2	3	1e-21	*Urechis caupo*
CB174588	40S ribosomal protein S3a	2	1e-71	*Brassica rapa*
CB174589	40S ribosomal protein S4	2	1e-35	*Dictyostelium discoideum*
CB174590	40S ribosomal protein S4	10	5e-41	*Zea mays*
CB174591	40S ribosomal protein S5	2	4e-46	*Theileria annulata*
CB174592	40S ribosomal protein S9	1	2e-34	*Podospora anserina*
CB174593	40S ribosomal protein S19	1	4e-036	*4e-036*
CB174594	40S ribosomal protein S23	1	4e-52	*Spodoptera frugiperda*
CB174595	40S ribosomal protein S28 (S33)	1	5e-11	*Kluyveromyces marxianus*
CB174596	40S ribsomal protein S29	1	1e-14	*Saccharomyces cerevisiae*
CB174597	60S ribosomal protein L3	5	5e-71	*Arabidopsis thaliana*
CB174598	60S ribosomal protein L7	2	1e-35	*Cyanophora paradoxa*
CB174599	60S ribosomal protein L7a	5	9e-40	*Arabidopsis thaliana*
CB174600	60S ribosomal protein L10	7	3e-82	*Drosophila melanogaster*
CB174601	60S ribosomal protein L10a	25	5e-32	*Mus musculus*
CB174602	60S ribosomal protein L13	2	6e-20	*Arabidopsis thaliana*
CB174603	60S ribosomal protein L14	1	3e-16	*Chara globularis*
CB174604	60S ribosomal protein L23	6	1e-49	*Phytophthora infestans*
CB174605	60S ribosomal protein L18a	1	1e-030	*Phytophthora infestans*
CB174606	60S ribosomal protein L27a	3	1e-065	*Phytophthora infestans*
CB174607	60S ribosomal protein L29	2	3e-13	*Panax ginseng*
CB174608	60S ribosomal protein L35	1	1e-11	*Gallus gallus*
CB174609	60S ribosomal protein L37a	2	4e-017	*Phytophthora infestans*
CB174610	60S ribosomal protein L44	1	8e-24	*Caenorhabditis elegans*
CB174611	Ribosomal protein	2	7e-09	*Phytophthora infestans*
CB174612	Ribosomal protein	2	7e-11	*Phytophthora infestans*
CB174613	Ubiquitin/ribosomal protein S27a	4	4e-05	*Acanthamoeba castellanii*
CB174614	Ubiquitin/ribosomal fusion protein	3	2e-43	*Schizosaccharomyces pombe*
CB174615	20S proteasome subunit alpha 1	1	2e-15	*Nicotiana tabacum*
CB174616	20S proteasome subunit alpha 4	1	3e-15	*Arabidopsis thaliana*
CB174617	20S proteasome subunit beta 7	1	6e-20	*Danio rerio*
CB174618	26S proteasome subunit 4 ATPase	1	9e-53	*Arabidopsis thaliana*
CB174619	tef1 elongation factor	78	1e-105	*Phytophthora infestans*
CB174620	Initiation factor 5A-3 (eIF-5A 3)	1	2e-23	*Arabidopsis thaliana*
CB174621	Possible Myb_DNA-binding protein	1	2e-008	*Phytophthora infestans*
CB174622	BTF3-like	3	5e-20	*Oryza sativa*
CB174623	FK506-binding protein	1	1e-029	*Tetrahymena thermophila*
CB174624	Cyclophilin b	3	5e-62	*Dictyostelium discoideum*
**Signal transduction**				
CB174625	Serine/threonine kinase	1	3e-10	*Nostoc sp. PCC 7120*
CB174626	Phosphoinositide phospholipase C	1	7e-66	*Solanum tuberosum*
CB174627	Pyrophosphatase	1	2e-57	*Phytophthora infestans*
CB174628	Calmodulin	3	1e-43	*Pythium splendens*
CB174629	Calmodulin	1	3e-20	*Myxine glutinosa*
CB174630	G protein beta subunit	1	5e-10	*Chlamydomonas reinhardtii*
CB174631	GTP binding protein	2	9e-92	*Phytophthora infestans*
CB174632	Annexin VII	1	1e-16	*Dictyostelium discoideum*
CB174633	Annexin VII	1	3e-13	*Artemia franciscana*
CB174634	Annexin VII	1	2e-10	*Dictyostelium discoideum*
CB174635	NADPH oxidoreductase	2	2e-33	*Nostoc sp. PCC 7120*
**Stress response**				
CB174636	Manganese superoxide dismutase	1	7e-069	*Phytophthora nicotianae*
CB174637	Glutathione transferase, theta class	2	4e-059	*Phytophthora infestans*
CB174638	Heat shock protein 70	3	2e-070	*Phytophthora nicotianae*
CB174639	Glucose regulated protein/BiP	4	1e-52	*Phytophthora cinnamomi*
CB174640	hsp 70	3	5e-52	*Rimicaris exoculata*
CB174641	hsp 70	1	8e-26	*Mitsukurina owstoni*
CB174642	hsp 90	1	6e-06	*Eimeria tenella*
CB174643	Copper chaperone	1	6e-08	*Plantago major*
CB174644	Thioredoxin peroxidase	1	1e-102	*Phytophthora infestans*
CB174645	Nucleoredoxin	1	2e-07	*Mus musculus*
**Elicitor and pathogenicity**				
CB174646	Transglutaminase elicitor M81E	2	1e-15	*Phytophthora infestans*
CB174647	Pectin methylesterase	1	1e-23	*Cochliobolus carbonum*
CB174657	Kazal-like serine protease inhibitor EPI9	1	2e-09	*Phytophthora infestans*
CB174713	Cystatin-like Cysteine proteinase inhibitor	1	2e-42	*Phytophthora infestans*
**Hypothetical proteins**				
CB174648		3	1e-28	*Mus musculus*
CB174649		3	3e-16	*Streptomyces coelicolor*
CB174650		4	2e-15	*Drosophila melanogaster*
CB174651		1	2e-14	*Magnetococcus sp. MC-1*
CB174652		2	4e-13	*Mus musculus*
CB174653		1	5e-10	*Vibrio cholerae*
CB174654		1	7e-06	*Schizosaccharomyces pombe*
CB174655		1	5e-06	*Glycine max*
**p-value > e-05**				
CB174656, CB174658 to CB174712		95*		

(a) Contains also the primers sequences used in this study.

(b) Species with which the BlastX homology was observed.

### Functional classification of *P. halstedii *ESTs

ESTs were assigned to putative cellular roles using the categories defined by Bevan *et al.*[19] and the Expressed Gene Anatomy Database (EGAD) [20]. Two categories (elicitor and pathogenecity, and cell defence) are added as described by Kamoun *et al.*[21]. A summary of the assignment of non-redundant ESTs to functional categories as well as their relative abundance is listed in Table 3. The majority of the identified cDNAs were related to protein synthesis, cell metabolism, signal transduction, and cell stress.

### Protein-signature scanning and identification of putative secreted proteins

Because a large set of the ESTs (64) were predicted to code for hypothetical proteins or showed no significant homology with known proteins in the databases, we used SignalP 3.0 [22] to identify potential secreted proteins among this set. Additional information on these ESTs was obtained by protein-signature scanning. InterProScan was used for sequence comparison to the InterPro database [23]. The results of these searches are summarized in Table 4. The majority of these ESTs (47) did not display any reported motif or a signal peptide. Among those which displayed significant protein-signatures, five are predicted to contain a signal peptide, thus they may correspond to secreted proteins (Table 4). However, the average size of the ESTs was about 500 bp which is too small to allow the identification of a larger number of potentially secreted proteins.

**Table 4:** InterProScan search for motifs within the hypothetical proteins *of P. halstedii*

**GenBank: Accession number**	**InterProScan signatures**
CB174681	Aminoacyl-tRNA synthetase, class I
CB174654; CB174706	Endoplasmic reticulum targeting sequence
CB174653	Glycine cleavage H-protein
CB174649	Phosphotransferase system, HPr histidine phosphorylation site
CB174674	Plant metallothionein, family 15
CB174651	Purine phosphorylase, family 2
CB174650; CB174684; CB174698CB174658; CB174697	Signal-peptide
CB174679	Sperm-activating peptide
CB174648	TB2/DP1 and HVA22 related protein
CB174659	Trensmembrane-region

### Identification of pathogenicity-related genes

**Figure 2:**
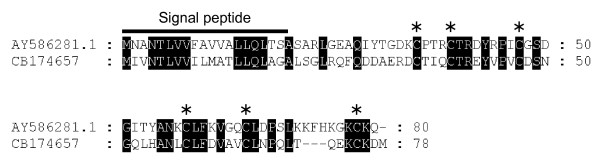
**Alignment of Kazal-like proteins from *P. infestans *and *P. halstedii***. One Kazal-like protein from *P. halstedii *[GenBank:CB174657] and the EPI9 Kazal-like protein from *P. infestans *[GenBank:AY586281.1] were aligned using ClustalX program. The asterisks indicate the conserved cysteine backbone defining the Kazal-like proteins family. The potential Peptide signal is also indicated.

Functional annotation of the ESTs identified at least 4 that could be potentially involved in the infectious process of *P. halstedii*. One EST [GenBank:CB174657] showed homology with a Kazal-like serine protease inhibitor from *Phytophthora infestans *[24]. Alignment of these sequences (Figure 2) shows that the *P. halstedii *Kazal-like protein contains the conserved cysteine backbone and the motif C-X3-C-X7-C-X10-C-X6-C-X9-C defining the Kazal family signature [24].

**Figure 3:**
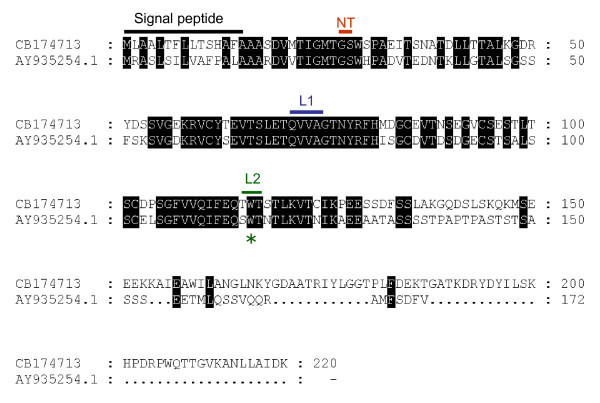
**Alignment of Cystatin-like proteins from *P. infestans*and *P. halstedii***. The *Phytophthora infestans *Cystatin-like protein EPIC4 [GenBank:AY935254] was aligned with the putative *P. halstedii *Cystatin-like protein [GenBank:CB174713]. NT indicates the N-terminus trauncation, L1 and L2 indicate two conserved loop. The asterisk indicates the conserved Tryptophane amino acid within the L2 loop. The potential Peptide signal is also indicated.

A second EST [GenBank:CB174713] showed a strong homology with a *Phytophthora infestans *Cystatin-like protein [25]. The *P. halstedii *cystatin-like protein displays 42% identity with the *P. infestans *cystatin-like protein (Figure 3). Interestingly, as in the *P. infestans *protein, the *P. halstedii *protein contains a potential signal-peptide and conserved domains, including the N-terminal trunk (NT), first binding loop (L1) and second binding loop (L2) [25], suggesting that these proteins may have similar functions.

### Comparison with true fungi and other microbes for conserved virulence factors

The PHI-Base is a database containing expertly curated molecular and biological information on genes proven to affect the outcome of pathogen-host interactions [26]. Blastx search of this database identified 11 out of the 145 *P. halstedii *ESTs with significant similarity (E value < E^-5^) (Table 5). The matching hits belong both to plant and animal pathogens. Interestingly, 6 out of the eleven genes are required for pathogenicity on animals. The homologous genes are involved in a variety of cellular functions such as signal transduction (PHI:221) or cellular detoxification (PHI:410).

**Table 5:** Homology search with virulence factors in the PHI-base database

***P. halstedii *(GenBank: Accession N)**	**PHI Hits (a)**	**BlastX E-Value**	**Function (b)**
CB174618	PHI:593 *Magnaporthe*	6e-08	Triphosphatase activity
CB174624	PHI:249 *Magnaporthe*	2e-42	Isomerase activity
CB174646	PHI:39 *Phytophthora*	6e-17	Glutamyltransferase activity
CB174647	PHI:278 *Botrytis*	9e-22	Pectinesterase activity
CB174623	PHI:548 *Botrytis*	2e-19	Protein folding
CB174628	PHI:474 *Cryptococcus*	1e-12	Calcium ion binding
CB174636	PHI:410 *Cryptococcus*	1e-25	Superoxide dismutase activity
CB174644	PHI:386 *Cryptococcus*	3e-52	Peroxidase activity
CB174570	PHI:504 *Saccharomyces*	1e-16	Leucine biosynthesis
CB174578	PHI:624 *Salmonella*	4e-21	ATPase activity
CB174625	PHI:211 *Candida*	7e-08	Regulation of transcription

(a) The PHI hits indicate the accession numbers at the PHI-base database [25] and the corresponding species.

(b) The function of the gene as indicated in the PHI-base database. See the references therein for more details.

### Comparison with other Oomycetes

Search for homologous sequences in the ESTs collections or whole genomes sequences of different oomycetes showed that 117 out of the 145 *P. halstedii *ESTs share similarity with sequences in at least one oomycete taxon with a BlastN E-value < E^-5^. When an E-value cutoff < E^-20 ^is used, 82 *P. halstedii *ESTs still shared similarity with sequences in the other oomycetes. Neverthless, 11 *P. halstedii *showed no significant homology with any sequence in the other oomycetes or any other sequence in the public databases. Thus, these sequences are unique to *P. halstedii *and may represent genes specific to this pathogen. Unfortunately, these 11 ESTs are annotated as hypothetical and no obvious motif was detected within them using Interproscan and Smart programs.

Fifteen sequences were highly similar (BlastN E-value < E^-20^) with sequences in at least 5 out of the 8 oomycetes taxa. These sequences included 3 ESTs coding for unknown proteins ([GenBank:CB174649], [GenBank:CB174661] and [GenBank:CB174701]) and 12 ESTs coding for differents proteins such as calmodulin [GenBank:CB174628], actin [GenBank:CB174582] or ribosomal proteins ([GenBank:CB174588] and [GenBank:CB174589]).

## Discussion

Many plant diseases are caused by parasitic microorganisms for which little molecular information is available. Thus, the large scale sequencing of Expressed Sequence Tags (EST) could be considered as a first step towards understanding the molecular basis of pathogenicity of these microorganisms. This approach allows rapid and exhaustive sampling of transcripts that are regulated during the infection process. For example, 704 unisequences have been identified in the wheat pathogen *Mycosphaerella graminicola *(*Septoria tritici*) [27]. In this study, we were interested in the identification of transcripts produced by *P. halstedii *during the infection of its host. However, a major challenge is the biotrophic nature of this parasite. To overcome this limitation, we decided to use the suppression subtractive hybridization (SSH) method [16]. One of its main advantages is that it allows the detection of low-abundance differentially expressed transcripts, such as many of those likely to be involved in signal transduction.

### Redundancy

The 230 unisequences correspond to 153 clones present as singletons and 398 clones corresponding to redundant cDNA which formed 77 contigs ranging from 2 to 78 ESTs. This redundancy rate of 72% is higher than those obtained from other EST sequencing programs, for example, 49% of 1409 clone of *N. crassa *[28], 53% of 4809 clones from the cambial tissue of poplar [29], and 37% of 1000 clones from *Phytophthora infestans *[21]. This redundancy rate of 72% could be reduced by sequencing more clones after a differential screen with the most represented clones, elongation factor of *P. halstedii *and Asparagine synthase of *Heliantus annuus *which were represented in 78 and 56 copies respectively and account for up to a third of redundancy observed.

### Origin of the EST

The 230 unisequences may correspond either to sunflower genes induced upon the infection by *P. halstedii *or sequences originating from the parasite. To overcome this difficulty, 230 primer pairs were designed and used to amplify the corresponding fragment with sunflower and *P. halstedii *DNA. This analysis resulted unambiguously in the identification of 145 EST originating from *P. halstedii *which corresponds approximately to 63% of the total of unisequences. The sequences of these primers are deposited along with the sequences of the ESTs in the Genbank. When the EST belongs to *P. halstedii*, amplification product was observed only when the DNA of *P. halstedii *extracted from infected sunflowers is used as template. Conversely, when the EST is originating from sunflower, a faint amplification product is often observed with DNA from *P. halstedii *(Figure 1). This is due to the contamination of sporangia with sunflower cells when being collected from infected cotyledons. Although this PCR strategy is robust, it is not suitable for high throughput sequencing project. Alternatively, in a similar work, Thara *et al. *[30] confirmed the fungal origin of several EST from the obligate basidiomycete *Puccinia tritici *by hybridization with radioactively labelled total genomic DNA. When the whole genome sequence of the host is available such as in the model plant *A. thaliana*, it can be exploited to distinguish between the EST originating from the host and those originating from the parasite. This strategy has been used by van der Biezen *et al.*[14] to identify 7 genes from the oomycete *Peronospora parasitica*. The G+C content of the EST also has been used to distinguish *Phytophthora sojae *from soybean cDNA [31]. The average G+C content of soybean EST was 46% whereas the G+C content of *Phytophthora sojae *was 58%, and plotting of the ESTs from infected soybean produced two distinct peaks of G+C percentage [31]. In the present study, the percentage G+C contents of ESTs from *P. halstedii *and from sunflower were entirely overlapping with averages of 47% and 45% respectively (data not shown), making this criterion inappropriate to uncover the origin of an EST in this pathosystem.

In contrast, all the sequences showing homology with sequences from other oomycetes such as *Phytophthora *species proved to be originating from *P. halstedii*. Therefore, the growing number of sequences produced in different oomycete EST sequencing projects and the availability of *Phytophthora *and *H. parasitica *genome sequences should facilitate the analysis of pathogen genes expressed during host-dependent stages.

### Functional classification of *P. halstedii *ESTs

Many of the ESTs identified in this study are associated with basic metabolisms such as energy production, carbohydrates metabolism, nucleotides and protein synthesis. Protein synthesis process is highly represented which may indicate that this process is actively involved during infection. The most represented EST [GenBank:CB174619] shows significant similarity with the tef1 elongation factor from *Phytophthora infestans *[32], which is highly expressed during spore germination and mycelium formation [33]. Interestingly, this gene has affinity for actin and tubulin and may be involved in the regulation of the cytoskeleton [33].

The EST [GenBank:CB174624] shows homology with cyclophilin which has a cis-trans isomerase activity [34]. However, this gene has also been identified as a virulence factor in the rice blast fungus, *Magnaporthe grisea *[35]. It should be interesting to test whether this gene has conserved functions in different plant pathogen species as it was hypothesized by Thara et al. [30].

The homology found between one EST [GenBank:CB174646] and an elicitor from *P. megasperma *[36] indicates that the EST approach can be a rapid way to generate sequences potentially involved in the pathogenicity process. However, whether this gene has a similar function in *P. halstedii *has still to be experimentally demonstrated. Additionally, many ESTs share homology with stress-related genes such as superoxide dismutase [GenBank:CB174636] or gluthatione peroxidase [GenBank:CB174637] which may indicate that *P. halstedii *faces a hostile environment within the sunflower tissues and that such enzymes may detoxify compounds released by the host such as hydrogen peroxide. For example, in *Mycobacterium tuberculosis*, a superoxide dismutase enzyme contributes to the resistance of the parasite to the oxidative burst in macrophages [37].

### Identification of *P. halstedii *genes potentially involved in the pathogenesis process

Two of the ESTs obtained share significant homology with protease inhibitors from *P. infestans*. The first one [GenBank:CB174657] contains a Kazal-like domain and is similar to the serine protease inhibitor EPI9 from *P. infestans *[24]. At least 35 Kazal-like Serine protease inhibitors have been reported in different oomycetes and two of these secreted proteins (EPI1 and EPI10) have been shown to interact and inhibit the apoplastic pathogenesis-related Protease P69B, a subtilisin-like serine protease of tomato [24,38]. Interestingly, Catanzariti et al. [39] showed that the flax rust avirulence gene *AvrP123-A *encodes a Kazal-like protein that is recognized by *P1 *and *P2 *resistance genes in flax. The putative *P. halstedii *Kazal-like protein possesses all the conserved domains defining the Kazal-like family. The second *P. halstedii *protease inhibitor like protein [GenBank:CB174713] shares similarity with a *P. infestans *Cystatin-like protein [38]. Both sequences possess all the signatures sequences of the Cystatin-like protease inhibitors, including the N-terminal trunk, the first loop and the conserved Trp within the second loop. It is likely that these sequences similarities may reflect conserved physiological functions. Thus, it should be interesting to test experimentally whether theses proteins could similarly inhibit proteases of infected sunflowers.

### Identification of potentially shared factors with true fungi

We exploited the recently developed PHI-base [40] a database that catalogues the phenotypes resulting from mutations in defined genes of both plant and animal pathogens [26]. Homology search using the *P. halstedii *ESTs identified 11 sequences with significant matches in this database. Mutation of the identified genes led to reduced virulence in the respective hosts. For instance, the *P. halstedii *[GenBank:CB174644] is highly similar to a thiol peroxidase (PHI:386) from the basidiomycetous fungus *Cryptococcus neoformans*. As peroxidases, this gene acts to remove peroxides and provide defence against oxidative damage. Mutation of this gene significantly reduced virulence in mice [41]. We have shown that resistance of sunflower to *P. halstedii *is associated with an oxidative-like burst within the hypocotyls [3]. Thus, it should be interesting to test whether the *P. halstedii *thiol peroxidase gene plays a similar role in detoxifying peroxides in sunflower. Sequence homology does not necessarily imply a conserved function, yet many animal and plant pathogens appear to utilize common signaling cascades and protective compounds during their development and pathogenesis [42].

## Conclusion

In this study we have initiated an EST approach combined with SSH PCR to obtain for the first time genes from the mycelium of the obligatory oomycete *P. halstedii*.

Nevertheless, in the long-term process towards the identification of pathogenicity factors in *P. halstedii*, it should be necessary to test the physiological function of each EST by genetically transforming *P. halstedii in planta*, as it was developed for *Erisyphe graminis f.sp*. *hordei *[43]. Recently, a transient expression of the gfp protein has been reported in *P. halstedii *sporangia using electroporation and a mechanoperforation method [44]. However, the gfp expression was lost during the subsequent rounds of infection. Alternatively, it should be interesting to know to which extent the metabolic pathways are conserved among the oomycetes and whether the heterologous expression of *P. halstedii *genes in a transformable oomycete such as *P. infestans *is useful. Overall, these resources will greatly accelerate research on this important pathogen and could lead to novel perspectives for controlling the pathogenicity of sunflower downy mildew.

## Methods

### *P. halstedii isolate *and culture conditions

One isolate identified as the physiological race 300 of *P. halstedii *on sunflower differentials was used. This isolate was provided by Dr D. Tourvieille (INRA-Clermont-Ferrand, France). It has been collected from infected fields in the south of France in 1995 and maintained by asexual reproduction on the sunflower genotype Peredovick, susceptible to all known races of *P. halstedii *in a containment culture chamber [45]. Frequently, sunflower differentials are infected to assess the behaviour of the isolate. The infection method of sunflower germinated seeds and growing conditions were those described by Mouzeyar *et al.*[2].

### Preparation of mRNA and Suppression Subtraction Hybridization library construction

Total RNA was extracted from 15-day-old infected and non-infected sunflower plants using the method described by Bogorad *et al.*[46] and the polyadenylated mRNA with the PolyATract mRNA Isolation System (Promega France). Using the Clontech PCR-select™ cDNA Subtraction kit [16], second-strand cDNAs are prepared from the two mRNA populations under comparison. To enrich the cDNA library with clones specifically from *P. halstedii*, the cDNA from infected plants was used as tester and the cDNA from non infected plants was used as driver. After subtraction, the PCR-amplified cDNA were cloned into pGemT-easy vector and transformed into *E. coli *JM 109 strain (Promega France).

### Sunflower and *P. halstedii *DNA extraction

Sunflower DNA was extracted from healthy young leaf tissues by Nucleon PhytoPure kit (Amersham™). To extract *P. halstedii *DNA, 15-day-old infected seedlings were placed in a plastic bag for 2 days to induce sporulation on cotyledons and leaves. Zoosporangia were collected by gently washing out the cotyledons and leaves with sterile water and DNA extracted using the Nucleon PhytoPure kit (Amersham™ France).

### DNA sequencing

Clones from the subtracted library were selected randomly and grown in 96-well plates. Each EST was single-pass sequenced using the Dye-Terminator method and the T7 primer (Genome Express, France).

### Sequence analysis

Sequences with low quality bases (Phred score less than 20) and short sequences (< 100 nucleotides) were removed from further analysis. The vector and polylinker sequences were manually trimmed. Sequences were then assembled and arranged into unisequences using the BlastN algorithm [17] and CAP3 program [18]. Similarity searches were done using the BlastX program [17] against current version of NCBI "nr" non-redundant amino acid database.

### Comparison with true fungi and other microbes for conserved virulence factors

The PHI-base containing the description of curated genes involved in pathogenicity both in animals and in plants were searched for homology with the *P. halstedii *ESTs [26]. The version 2.31 containing 592 proteins was downloaded and used in BlastX search using the BioEdit package v7.0.8 [47]. The hits with an E-value < E^-5 ^were considered as significant.

### Primers design and PCR amplification

Primer pairs were designed using the primer3 program [48] and used to amplify each EST from DNA isolated from *P. halstedii *or from DNA isolated from sunflower line. The PCR amplifications were carried out with 50 ng DNA in the presence of 0.2 mM of each dNTP, 1 U of *Taq *DNA polymerase (Advantage 2, Clontech), 1 × *Taq *polymerase buffer and 0.5 μM of each primer. PCR was carried out in a 9600 Perkin-Elmer thermocycler under the following conditions: 35 cycles of 10 s at 94°C (denaturation), 30 s at 60°C (primer annealing), and 1 min 30 s at 72°C (primer extension). PCR products were separated using standard TAE agarose gel electrophoresis.

## Authors' contributions

MFB, FP and SM, performed the experiments including construction of the cDNA library, annotation and analyses of the sequences. MFB, PN and SM wrote the manuscript. All authors read and approved the final manuscript.
